# The world’s first digital cell twin in cancer electrophysiology: a digital revolution in cancer research?

**DOI:** 10.1186/s13046-022-02507-x

**Published:** 2022-10-11

**Authors:** Christian Baumgartner

**Affiliations:** Institute of Health Care Engineering With European Testing Center of Medical Devices, Computational Cancer Electrophysiology Lab, Graz University of Technology, 8010 Graz, Austria

**Keywords:** Digital cancer cell twins, In-silico models, Cancer electrophysiology, A549 cells, Human lung adenocarcinoma

## Abstract

**Background:**

The introduction of functional in-silico models, in addition to in-vivo tumor models, opens up new and unlimited possibilities in cancer research and drug development. The world's first digital twin of the A549 cell's electrophysiology in the human lung adenocarcinoma, unveiled in 2021, enables the investigation and evaluation of new research hypotheses about modulating the function of ion channels in the cell membrane, which are important for better understanding cancer development and progression, as well as for developing new drugs and predicting treatments.

**Main body:**

The developed A549 in-silico model allows virtual simulations of the cell’s rhythmic oscillation of the membrane potential, which can trigger the transition between cell cycle phases. It is able to predict the promotion or interruption of cell cycle progression provoked by targeted activation and inactivation of ion channels, resulting in abnormal hyper- or depolarization of the membrane potential, a potential key signal for the known cancer hallmarks. For example, model simulations of blockade of transient receptor potential cation channels (TRPC6), which are highly expressed during S-G2/M transition, result in a strong hyperpolarization of the cell’s membrane potential that can suppress or bypass the depolarization required for the S-G2/M transition, allowing for possible cell cycle arrest and inhibition of mitosis. All simulated research hypotheses could be verified by experimental studies.

**Short conclusion:**

Functional, non-phenomenological digital twins, ranging from single cells to cell–cell interactions to 3D tissue models, open new avenues for modern cancer research through "dry lab" approaches that optimally complement established in-vivo and in-vitro methods.

## Background

With the increasing availability of experimental data in cancer electrophysiology using patch clamp and fluorescence imaging techniques in recent years, and the established knowledge of mathematical modeling and computer simulation of excitable cells up to the whole organ such as the heart [1], the era of digital twins in cancer research has also been ushered in. Since functional in-silico cell models have been standard tools in biomedical research and application for many years, especially in cardiac or neural electrophysiology, some 70 years after the first publication of an ion current model of a nerve cell by Hodgkin & Huxley in 1952, the first in-silico whole cell model in cancer electrophysiology was published in 2021 [2]. This has provided an important foundation for functional cancer cell models that are now available for the first time in addition to classical in-vivo tumor models such as cell line-derived xenograft, patient-derived xenograft or syngeneic models to simulate cellular mechanisms of cancer development and progression including cell differentiation, proliferation, migration and apoptosis.

Digital twins are usually based on mathematical descriptions of the underlying cellular and intercellular mechanisms of the biological system and represent a mirror image of the real system at different levels of abstraction. However, most of these in-silico cell models used in cancer research are phenomenological, descriptive in nature and lack a detailed mathematical description of the biological mechanisms. These so-called phenomenological models, known as discrete, agent-based, or individual-based models, can simulate tumor growth, the generation of tumor heterogeneity, interactions within tumors, or predicting treatment outcome, but with limitations, as they primarily reflect observations of the biological mechanisms under study by attempting to capture simple mechanistic explanations for patterns in the data collected [3–7]. In turn, the first digital twin in cancer represents a functional description of the *electrophysiological system* of a cancer cell, and thus forms an essential basis for future in-silico tools in cancer research.

There are three reasons why functional cell models have not been introduced earlier: First, due to the phenotypic and functional heterogeneity of cancer cells within the same tumor as a consequence of genetic changes, environmental differences and reversible changes in cell properties, the large number of different types of cancer and their manifestations, and the limited availability of extensive experimental data, it has been nearly impossible to develop functional digital twins of cellular and intercellular mechanisms including tumor heterogeneity and cancer cell plasticity.

Second, the wide availability of advanced mathematical models of excitable cells up to complete organ models as in cardiac electrophysiology has already led to first digital twins even of the whole organ [1]. This is not the case in cancer due to its high phenotypic and functional diversity.

Third, the not fully understood electrophysiological mechanisms of non-excitable cancer cells during the cell cycle have hindered the introduction of functional models in cancer electrophysiology in the past. Stronger depolarization of the cell membrane potential in various cancers is a well-known feature, but its modulation during the cell cycle has not been fully studied. Therefore, perturbations of ion channel regulation caused by various physical, biochemical, or biological stimuli of the cell microenvironment or by external pharmacological stimuli can be thoroughly investigated or even controlled with such computer models.

## Main text

Cancer cell genotypes are generally expressed by multiple pathophysiological factors associated with manifestations of the known cancer hallmarks [8]. Cancer-affected genes may encode ion channels responsible for maintaining intracellular ionic homeostasis and signaling within the cell and among cells, and contribute to the pathophysiological features of each cancer hallmark to varying degrees [9]. As mammalian cells express a large number of structurally and functionally distinct ion channels (Na^+^, K^+^, Ca^2+^, Cl^−^) in the cell plasma membrane and in the intracellular membranes of organelles, cancer cells are able to modulate the membrane potential through abnormal depolarization and hyperpolarization during the cell cycle, caused by perturbations in the regulation of the channels function and the control of important signaling mechanisms. However, compared to excitable cells, the potential changes occur very slowly and throughout the cell cycle over hours and days, and may serve as a key signal to trigger or prevent transition between the cell cycle phases. Therefore, a better understanding of the underlying electrophysiological mechanisms will enable new efforts for targeted treatment of cancer [10].

**Fig. 1:**
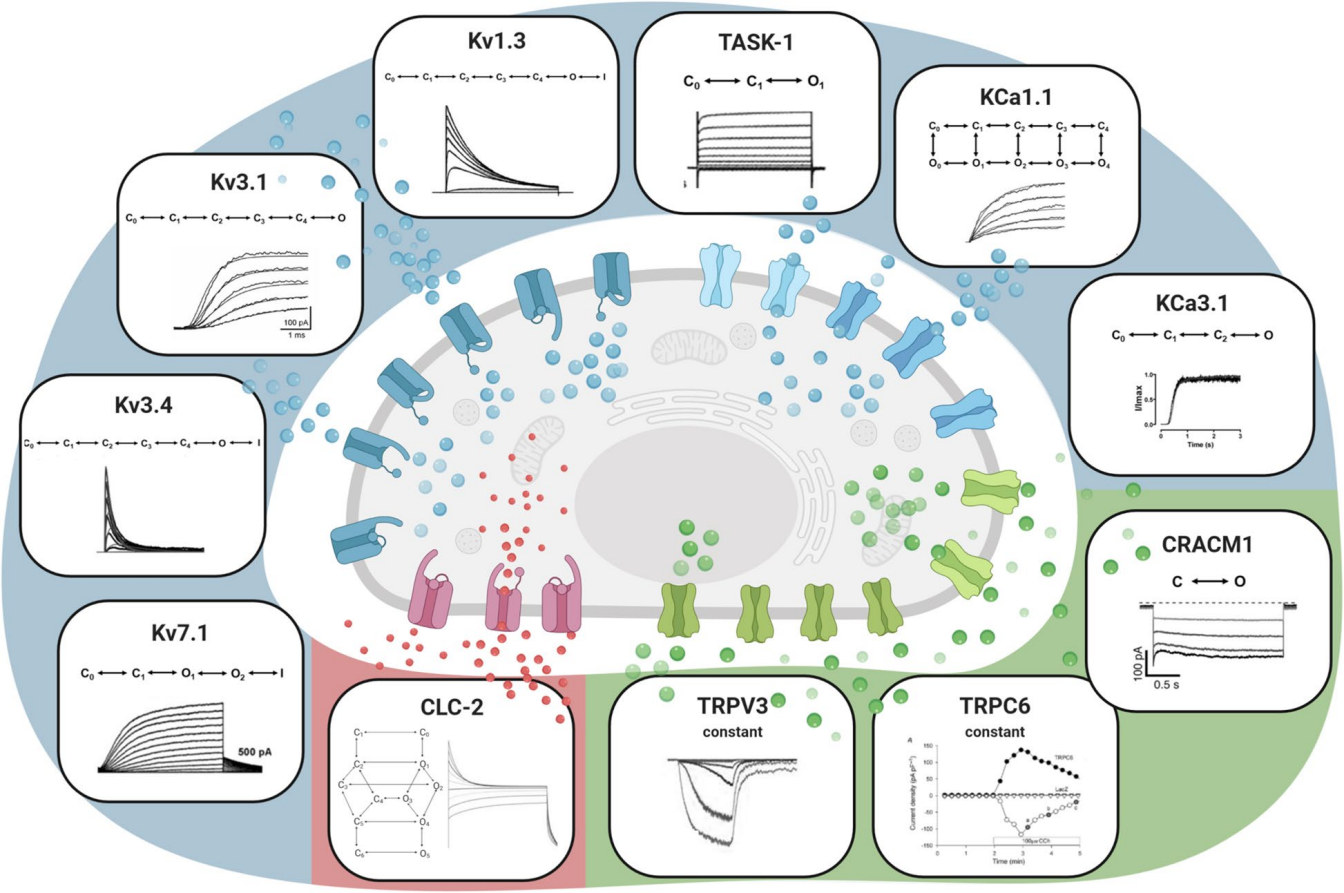
A549 whole-cell ion current model, illustrating the different ion channel types, macroscopic currents and kinetic schemes of the used hidden Markov models (HMM). Potassium channels, including Kv1.3, Kv3.1, Kv3.4, Kv7.1, TASK-1 KCA1.1 and KCa3.1 are represented in blue, green denotes the included calcium channels CRACM1, TRPV3 and TRPC6 and red represents the considered chloride channel CLC2. This was taken from Langthaler S & Baumgartner C et al. [2]

**Fig. 2:**
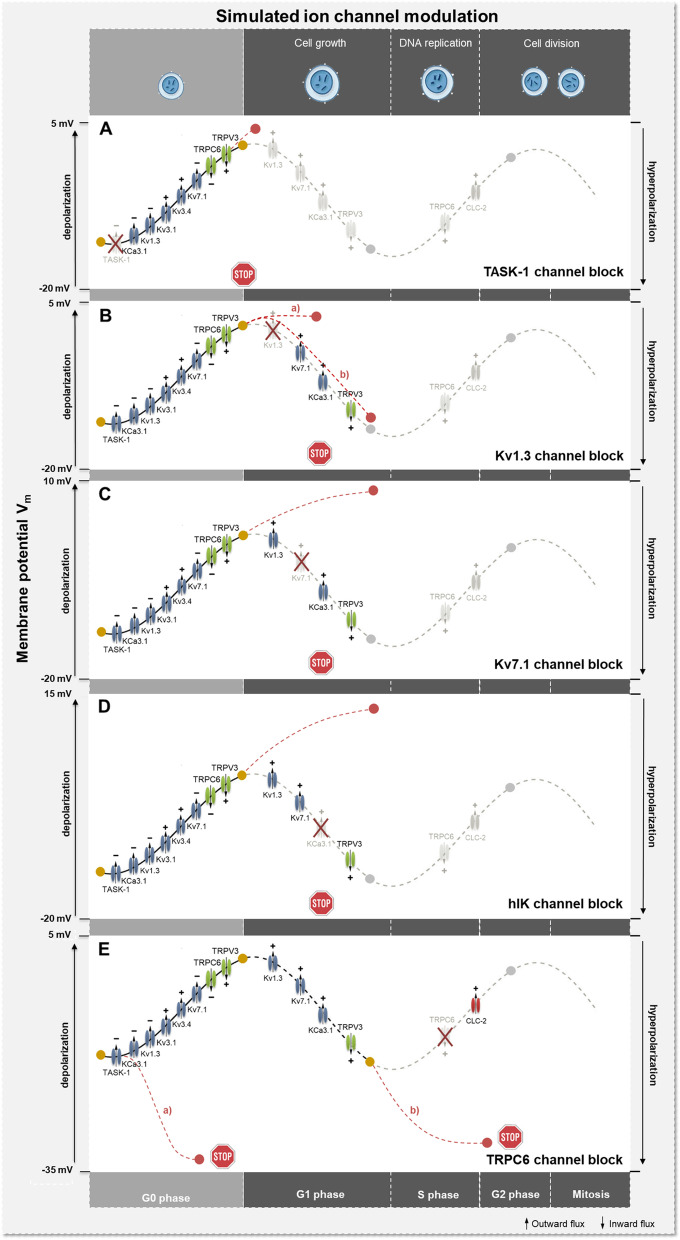
Schematic illustration of model simulations of ion channel activation and inactivation during cell cycle progression, resulting in abnormal depolarization or hyperpolarization of the membran potental. **A** Simulation of TASK-1 channel blockage in G1 phase. **B** Simulation of Kv1.3 channel blockage in a) G1 phase and b) during transition from G1 to S phase. **C** Simulation of Kv7.1 channel blockage starting at G1 phase. **D** Simulation of KCa3.1 channel blockage during G1-S transition. **E** Simulation of TRPC6 channel blockage in a) G0 phase and b) during transition from S to G2/M phase. The symbol + means activation,—means inactivation of ion channels. This was taken from Langthaler S & Baumgartner C et al. [2]

The recently introduced A549 in-silico model can now be used to simulate and predict the oscillatory changes in the membrane potential during the cell cycle (Fig. 1). Modulation of the potential at different cell cycle stages can lead to promotion or interruption of cell cycle progression by usually stronger hyper- or depolarization and can be induced by targeted activation and inactivation of ion channels. The TASK-1 channel, for example, is a proven A549 cell cycle modulator and its inhibition results in depolarization of the cell membrane associated with reduced proliferation, mitosis and enhanced apoptosis. Reduction or even blockage of TASK-1 channel activity in G1 phase leads to depolarization of the membrane potential, as confirmed by the model simulations, and could arrest cells in G1 phase, preventing proliferation. Inhibition of Kv1.3 channels was shown to be associated with depolarization of the cell membrane potential, accompanied by cell cycle arrest by impeding G1-S transition in A549 cells. Recent studies have shown that Margatoxin (MgTX), a specific Kv1.3 channel blocker, reduces proliferation and suppress tumor growth of A549 lung adenocarcinomas in-vivo. Simulation of inhibition of the Kv1.3 current in G1 phase results in little additional depolarization of the membrane potential, confirming this observation. TRPC6 channels, on the other hand, are highly expressed during S-G2/M transition. Inhibition of these channels results in arrest of the cell cycle and decreased mitosis, invasion and proliferation. Simulating blockade of TRPC6 channels in S phase leads to a strong hyperpolarization of the estimated membrane potential, which can suppress or bypass the depolarization required for the S-G2/M transition and allow cell cycle arrest and inhibition of mitosis (see Fig. 2) [2].

## Conclusions

With the first functional, non-phenomenological digital cell twins and their successors expected to exhibit higher levels of functional complexity, there is high potential to revolutionize modern cancer research through these new digital tools, which can range from single cell, to cell–cell interaction to 3D tissue models. In particular, the A549 digital twin of cell electrophysiology confirms that the changing membrane potential is a potential key signal for the known cancer hallmarks that should receive more attention in cancer electrophysiology research.

## Data Availability

Not applicable.

## Abbreviations

A549: Adenocarcinomic human alveolar basal epithelial cells (cell line)
HMM: Hidden Markov model
TASK-1: TWIK related acid-sensitive potassium channel
Kv1.3: Voltage-gated potassium channel
Kv3.1: Voltage-gated potassium channel
Kv3.4: Voltage-gated potassium channel
Kv7.1: Voltage-gated and lipid-gated potassium channel
KCA1.1: Calcium-activated potassium channel
KCa3.1: Calcium-activated potassium channel
TRPV3: Transient receptor potential cation channel
TRPC6: Transient receptor potential cation channel
CRACM1: Calcium-release-activated calcium channel
CLC2: Chloride channel
MgTX: Margatoxin, an alpha-KTx scorpion toxin
G0: Cellular state outside of the replicative cell cycle
G1: Gap 1 phase, or growth 1 phase
S: Synthesis phase
G2: Gap 2 phase, or growth 2 phase
M: Mitotic phase
